# A Longitudinal Examination of Real-World Sedentary Behavior in Adults with Schizophrenia-Spectrum Disorders in a Clinical Trial of Combined Oxytocin and Cognitive Behavioral Social Skills Training

**DOI:** 10.3390/bs12030060

**Published:** 2022-02-23

**Authors:** Julia Browne, Philip D. Harvey, Robert W. Buchanan, Deanna L. Kelly, Gregory P. Strauss, James M. Gold, Jason L. Holden, Eric Granholm

**Affiliations:** 1 Geriatric Research, Education and Clinical Center, Durham VA Health Care System, Durham, NC 27705, USA; 2 Department of Psychiatry and Behavioral Sciences, University of Miami Miller School of Medicine, Miami, FL 33136, USA; pharvey@med.miami.edu; 3 Research Service, Bruce W. Carter VA Medical Center, Miami, FL 33125, USA; 4 Maryland Psychiatric Research Center, Department of Psychiatry, University of Maryland School of Medicine, Baltimore, MD 21228, USA; rbuchanan@som.umaryland.edu (R.W.B.); dlkelly@som.umaryland.edu (D.L.K.); jgold@som.umaryland.edu (J.M.G.); 5 Department of Psychology, University of Georgia, Athens, GA 30602, USA; gstrauss@uga.edu; 6 Veterans Affairs San Diego Healthcare System, San Diego, CA 92161, USA; jlholden@health.ucsd.edu (J.L.H.); egranholm@health.ucsd.edu (E.G.); 7 Department of Psychiatry, University of California San Diego School of Medicine, San Diego, CA 92093, USA

**Keywords:** ecological momentary assessment, psychosocial intervention, functioning, physical health, serious mental illness

## Abstract

Sedentary behavior contributes to a shortened life expectancy in individuals with schizophrenia-spectrum disorders (SSDs), highlighting the need for effective interventions to improve health. This study examined whether reduced ecological momentary assessment (EMA) measures of sedentary activities were observed in individuals with SSDs who participated in a 24-week randomized trial of cognitive behavioral social skills training (CBSST) and either intranasal oxytocin or placebo (NCT01752712). Participants (*n* = 57) were prompted with EMA surveys seven times per day for seven days during the baseline, 12-week, and 24-week timepoints to sample sedentary behavior ratings, positive and negative affect, interpersonal interactions, and interpersonal interaction appraisals. Results revealed that sedentary behavior and social interactions did not significantly change over the 24-week clinical trial; however, positive and negative affect and defeatist interaction appraisals improved with treatment, and oxytocin produced modest additional improvements in these EMA outcomes. Greater momentary positive affect was significantly associated with greater activity and greater frequency of interactions. Overall, CBSST was effective at improving functioning, momentary affect, and defeatist interaction appraisals, although it did not reduce sedentary behavior; therefore, targeting these factors is not sufficient to reduce sedentary behavior, and adjunct interventions are needed.

## 1. Introduction

Due to physical health conditions, primarily cardiovascular disease, individuals with schizophrenia-spectrum disorders (SSDs) have a substantially shortened life expectancy compared to those without SSDs [1]. High levels of sedentary behavior in this population (up to 11 h/day) likely contribute to this poor health status and associated premature mortality [2,3,4]. In fact, a systematic review and meta-analysis of 47 studies found that increased sitting time was associated with a heightened risk of cardiovascular disease, type 2 diabetes, and all-cause mortality in the general population [5]. Moreover, being unable to get up out of a chair was the largest single predictor of unemployment beyond negative symptoms in a sample of persons with SSDs and bipolar illness [6], thereby contributing to disability in this population. As such, reducing sedentary behavior among individuals with SSDs is a critical first step in the facilitation of their improved health, quality of life, and survival. However, in order to adequately intervene and promote sustainable healthy behavioral changes it is important to consider the potential mechanisms underlying the high levels of sedentary behavior in this population.

The Ecological Momentary Assessment (EMA) represents a novel approach to exploring sedentary behavior and offers the potential to for insights into the mechanisms of excessive sitting in those with SSDs. EMA involves repeated assessments of momentary experiences (e.g., behaviors, thoughts, feelings) often administered multiple times a day through electronic surveys [7]. Unlike traditional objective (e.g., accelerometer) and subjective (e.g., retrospective self-reporting) measures of sedentary behavior that quantify the amount of time spent sitting more generally [8], EMA allows for reporting of specific engagement in activities in the moment, which can be categorized into sedentary and non-sedentary behaviors [9]. Furthermore, because EMA assesses momentary experiences, beliefs and emotions can be simultaneously collected to examine any relationships between sedentary behavior and emotional experience in order to identify potential motivations for sedentary behavior. This type of detailed analysis has the potential to extend prior work on sedentary behavior in those with SSDs by elucidating the subjective experience of excessive sitting time, which ultimately can inform the development of effective and sustainable interventions.

EMA has been effectively used with individuals with SSDs to measure clinical and functional outcomes such as positive and negative symptom severity and interpersonal interactions [10,11,12,13]. Additional EMA research has demonstrated that persons with SSDs spend greater amounts of time at home, engage in fewer reciprocal social interactions, and complete more nonproductive activities (e.g., watching television) than those without SSDs [14]. More recently, Strassnig and colleagues (2021) [15] re-analyzed data from the Granholm et al. (2020) study [14] to evaluate sedentary behavior in persons with SSDs compared to those without SSDs by categorizing the types of activities participants reported by movement and posture (i.e., recumbent, seated, standing, moving). The results showed that individuals with SSDs were more likely to engage in seated activity and less likely to perform moving or standing activities than those without SSDs [15]. Despite the greater amount of time engaged in seated and solitary activities, several EMA studies suggest that people with SSDs report greater positive affect when with other people compared to when spending time alone [16]. In terms of data on the locations of completed activities, our previous research using GPS geolocation collected with a mobile device suggested that people with SSDs manifested more negative affect when preparing to leave their homes and greater positive affect when on the way home compared to those without SSDs [17]. Taken together, this EMA work suggests that individuals with SSDs spend most of their time alone and engaged in sedentary activities; however, when engaged with others, particularly while at home, their mood is improved.

The potential reasons for high levels of solitary and sedentary behaviors may result at least in part from the difficulties with social cognition and social functioning commonly observed in this population [18]. Because of these challenges, individuals with SSDs may be reluctant to engage with other people due to low self-efficacy (e.g., “I’ll definitely fail”) and/or defeatist beliefs (e.g., “there’s no point in trying”) about performance during social interactions [19]. Further, negative symptoms such as avolition (i.e., lack of motivation) and asociality (i.e., lack of interest in social interaction) likely contribute to the high levels of sedentary and solitary behavior observed in those with SSDs [20]. Therefore, it may be that targeting negative symptoms and social challenges in those with SSDs can lead to increased interaction with others and a reduction in sedentary behaviors.

Cognitive behavioral social skills training (CBSST) is a group intervention that targets negative symptoms and social functioning in persons with SSDs by intervening on self-efficacy and defeatist performance beliefs [21]. CBSST has been rigorously evaluated and shown to be efficacious in improving functioning and reducing negative symptoms [19,22,23,24]. Considering its efficacy together with the potential for changes in these domains to lead to reduced sedentary behavior, we sought to explore the impact of CBSST on engagement in EMA-reported sedentary activities and the relationships with simultaneously-collected momentary measures of affect and interpersonal interaction appraisals. The central purpose of the present study was to explore longitudinal changes in real-world sedentary behavior of individuals with SSDs who participated in a clinical trial of CBSST and intranasal oxytocin or placebo [25]. The parent trial explored whether oxytocin, a neuropeptide involved in regulating social behavior, enhanced the effects of CBSST on overall psychopathology, negative symptoms, dysfunctional attitudes, and social functioning. Oxytocin was hypothesized to enhance social function based on evidence showing that oxytocin increases affect recognition and appraisal, trust, and theory of mind and reduces anxiety during interpersonal interactions [26,27,28,29]. Although results from the parent clinical trial revealed no added benefit of oxytocin on real-world social function or symptoms rated during in-person office visits [25], it is possible that the addition of oxytocin may have resulted in momentary changes in activities, moods/affect, interpersonal interactions, and/or interpersonal interaction appraisals, which could be captured by EMA measures.

The aims of this secondary analysis were to examine (1) changes in sedentary activities from baseline to 24-week endpoint; (2) changes in non-sedentary activities (i.e., those involving standing or movement) from baseline to 24-week endpoint; (3) changes in mood/affect, interpersonal interactions, and interpersonal interaction appraisals from baseline to 24-week endpoint; and (4) whether mood/affect was associated with engagement in sedentary and non-sedentary activities and whether interaction appraisals were associated with frequency of interactions on a longitudinal basis between baseline and 24-week endpoint. In addition, the impact of oxytocin on changes in sedentary and non-sedentary activities, mood/affect, interpersonal interactions, and interpersonal interaction appraisals was examined as an exploratory aim. Given that CBSST targets functioning and negative affective states, we expected that sedentary activities would decrease and non-sedentary activities would increase over the course of treatment and that these improvements would be associated with improvements in appraisals of interactions and decreased negative affect at these momentary assessments.

## 2. Materials and Methods

### 2.1. Study Design

The parent trial on which this secondary analysis is based was a 24-week, two-arm, double-blind, and placebo-controlled randomized controlled trial (RCT) with a 12-week post-treatment follow-up that tested CBSST plus intranasal oxytocin against CBSST plus placebo in a sample of adults with schizophrenia or schizoaffective disorder. The study was conducted at two sites: (1) the Maryland Psychiatric Research Center, University of Maryland School of Medicine and (2) the University of California, San Diego. This clinical trial was approved by the respective Institutional Review Boards and registered on clinicaltrials.gov (NCT01752712). Primary outcomes of the study have been published [25].

### 2.2. Participants

The inclusion criteria for the parent RCT were (1) age 18–55; (2) diagnosis of schizophrenia or schizoaffective disorder; (3) clinically stable; (4) in the nonacute phase of illness; (5) having a minimum level of social function impairment based on the asociality item of the Scale for the Assessment of Negative Symptoms [30]; and (6) if treated with a first- or second-generation antipsychotic medication, no change in dose for the past month or change in type for the past two months; the full exclusion criteria are available in [25].

The parent RCT included 62 participants that were randomized to one of the two treatment conditions (*n* = 31 in each arm). Groups did not significantly differ on age, sex, race, or education level (see Table S1 sample demographics).

### 2.3. Measures and Procedure

A full description of all study measures and procedures is available in the main outcome paper [25]. As the present analysis focused exclusively on EMA-measured variables, only these measures and the EMA procedure are described in detail here.

#### 2.3.1. EMA Procedure

Enrolled participants completed EMA assessments at baseline, mid-point (12 weeks), and endpoint (24 weeks). Participants were provided with a Samsung smartphone with Android OS to complete EMA surveys. EMA surveys were administered seven times per day for seven days during each of the assessment weeks. Signals occurred at stratified random intervals between 9:00 am and 9:00 pm each day; responses were only allowed within 15 min of the signal. Study staff were available for assisting with technology-related issues. Participants were not compensated for completing EMA surveys.

#### 2.3.2. EMA-Reported Activities

EMA surveys included questions about completion of 35 different activities that spanned the domains of leisure, treatment engagement, work/school involvement, self-care and hygiene, and household chores (Table 1). All questions about activities were asked about completion in the past hour (“In the past hour, did you [activity]”), with checkboxes for participants to select all activities that applied. Participants were asked to indicate how much time they spent at home in the previous hour [14].

#### 2.3.3. EMA-Reported Positive and Negative Moods/Affect

Each EMA survey included four items about mood/affect that covered ratings of positive affect (PA; happy, relaxed) and negative affect (NA; sad, nervous). Participants rated their experience of these four affective states on a scale from 1 (not at all) to 7 (extremely) at each survey. Ratings for happy and relaxed were averaged into a single metric of PA and ratings for sad and nervous were averaged into a single metric of NA.

#### 2.3.4. EMA-Reported Interpersonal Interactions and Appraisals

Surveys included questions about how many interactions participants had in the prior hour (with 0 as an option). If participants indicated they had any interactions, they were prompted to appraise their interpersonal interactions in the past hour by reporting their emotional experience (“how much warmth or trust did you feel for others in the interaction[s]?” and “how much pleasure or enjoyment did you feel in the interaction[s]?”), their communication competence (“how well do you think you communicated?”), and their perception of the other person’s opinion in the interaction (“what do you think others were thinking about you?”). All four interpersonal interaction appraisal items were rated on a 1–7 scale with different qualitative anchors: warmth/trust and pleasure (“not at all” to “very much”), communication competence (“terrible, failed, not worth trying” to “great, succeeded, worth the effort”), and perception of others’ opinion (“unlikeable, stupid or weird” to “likeable, smart, or interesting”).

### 2.4. Intervention

All participants received CBSST and were randomized to oxytocin or placebo. CBSST is a group-based psychosocial intervention that combines cognitive behavioral therapy and social skills training to teach communication skills and target interfering defeatist performance beliefs [21]. CBSST groups involve modeling and role-playing social situations to allow for practicing new skills and receiving positive and corrective feedback. Cognitive strategies to address interfering beliefs and problem-solving skills are discussed and employed during groups to overcome barriers to functioning. CBSST was delivered in four modules (Cognitive Skills, Social Skills, Problem-Solving Skills, and Social Cognition Skills) of six sessions each. The modules were delivered twice in order to compensate for cognitive impairment and to improve sense of mastery and self-efficacy, for a total of 48 sessions over the 24-week period.

Participants were randomized to one of two medication conditions: intranasal oxytocin (36 IU twice daily) or intranasal placebo. Participants received education and training on how to appropriately administer the medication. Study staff weighed intranasal medication bottles using an Ohaus AV313 Adventurer Pro scale to measure adherence.

### 2.5. Data Analysis

All analyses were conducted using SPSS version 27 (IBM, 2021) and all participants who completed the trial and responded to any EMA surveys were included (*n* = 57), regardless of EMA adherence.

Prior to analysis, the 35 EMA-reported activities were categorized into five activity domains: (1) recumbent, (2) seated at home, (3) seated away from home, (4) standing, or (5) moving (Table 1). These categories were established based on prior work [15] and on consensus among three members of the research team (JB, EG, PDH). Given that participants indicated whether or not they completed any of the 35 activities during each survey, a sum of all activities from each survey was calculated and the occurrence of each of the activity survey items was expressed as a proportion of all activities completed in each survey. Finally, the sums of the proportions of activities in each of the five categories was calculated.

Additionally, each participant was queried as to how many total interpersonal interactions they had had in the last hour and how those interactions were divided as between socially-focused (i.e., involving coworkers, classmates, friends, partners, and family) and non-socially-focused (i.e., involving treatment providers, staff, and roommates) interactions. The interpersonal interaction appraisal variables described above (warmth/trust, pleasure, competence, and others’ opinion) were only collected by the application for surveys where the number of interactions was greater than zero.

The data analyzed in this secondary analysis are longitudinal and consist of observations repeated seven times per day for seven days at three intervals separated by 12 weeks (baseline, weeks 12 and 24). For each of the five domains of activity (recumbent, seated at home, seated away from home, standing, moving), two interpersonal interaction variables (social and non-social), four interaction appraisals (warmth/trust, pleasure, competence, others’ opinion), and mood/affect (PA and NA) we performed a mixed-model repeated measures analysis of variance (MMRM ANOVA). We entered subject as a random intercept and used full information maximum likelihood methods to account for missing data. Site was entered as a covariate in all activity and interaction analyses. We only interpreted ANOVAs as significant if the omnibus statistic was significant, reflecting a significant improvement of the model over the null model. In exploratory analyses, models were re-run with the inclusion of treatment group (oxytocin or placebo) and the interaction of treatment group x treatment week.

In order to test whether changes in everyday activities were associated with improvements in interpersonal interaction appraisals or mood/affect states, we planned to use changes in PA (and/or NA, depending on the relationships between the variables) to predict changes in all five activity outcome domains, and planned to use changes in the four interpersonal interaction appraisal variables to examine any changes in their relationships with interpersonal interactions. This analysis was performed by entering the affect-related predictors into the analysis as a dynamic covariate.

## 3. Results

A total of 6622 EMA survey pages were scheduled, of which 4489 (68%) were answered. Adherence at all three timepoints ranged between 65 and 70 percent (baseline: 70%, week 12: 65%, week 24: 68%). Five activity variables and two interpersonal interaction variables were examined. At baseline, the most common types of activities were those done seated at home. During the past hour, participants reported approximately one social interaction. The baseline means for these activity domains and social interaction variables are presented in Table 2.

Each activity domain and interaction variable was predicted with the same time of day × day × week model following calculation of the omnibus test and random subject intercept. All seven omnibus tests were statistically significant (all *p* < 0.001). None of the weekly effects were statistically significant for any of the activity variables or for social interactions, and there were no significant two-way interactions involving week and day or time of day. Several of the activities manifested significant day or time of day effects, as would be expected (see Table S2 for these effects). The number of non-social interactions manifested a significant weekly effect, *X*^2^ (2) = 8.82, *p* = 0.012. However, the number of nonsocial interactions increased from 0.58 to 0.70 from baseline to week 12 and reverted to 0.58 interactions per survey at week 24. There were significant effects of site for the activities done seated at home (*X*^2^ (1) = 7.98, *p* = 0.005) and seated away from home (*X*^2^ (1) = 13.23, *p* < 0.001). The adjusted means for seated at home were 0.38 for San Diego and 0.41 for Maryland; for seated away, the adjusted means were 0.10 for San Diego and 0.13 for Maryland. There were significant effects of site for social (*X*^2^ (1) = 65.42, *p* < 0.001) and non-social (*X*^2^ (1) = 74.98, *p* < 0.001) interactions. The adjusted means for social interactions were 1.07 for San Diego and 1.53 for Maryland, while for non-social interactions the adjusted means were 0.85 for San Diego and 0.48 for Maryland. All other site analyses yielded *X*^2^ values less than 1.0.

The changes over the treatment trial for the affect and interpersonal interaction appraisals are presented in Table 3. Interaction appraisals were only collected when interpersonal interactions were reported in a survey; thus, the number of surveys was reduced to 3076 compared to the 4489 PA and NA surveys. Furthermore, the four interaction appraisal variables were very highly intercorrelated and all manifested the same treatment response; thus, we present only warmth/trust and competence. The omnibus tests were significant (all *p* < 0.003) for all four analyses (PA, NA, warmth/trust, and competence), as were the random subject intercepts (all *p* < 0.001). Week had a statistically significant effect on all four variables (all *p* < 0.001), with the effect reflecting increases in PA, as well as on improvement in appraisals of warmth/trust and competence and reduction in NA. Effects of day were all non-significant other than for warmth/trust (*X*^2^ (6) = 17.56, *p* = 0.007), and time of day effects were seen for two of the variables (PA: *X*^2^ (6) = 32.22, *p* < 0.001 and warmth/trust: *X*^2^ (6) = 12.89, *p* = 0.045), although these effects were much more modest than the weekly effects.

In order to examine whether mood/affect was associated with activities and whether interaction appraisals were associated with frequency of interactions, our next analysis was to recompute the MMRM analyses for the five activity domain outcomes while adding PA as a dynamic covariate, and to then recompute the analyses for social and non-social interactions with the average of all four interaction appraisal variables as the dynamic covariate. We entered PA only because the Pearson correlation between the aggregated PA and NA score was r = −0.52, *p* < 0.001. For all five of the activity analyses, the dynamic covariate of PA was significantly associated with activities, correlating positively for being seated away from home (*X*^2^ = 265.77, *p* < 0.001), standing, (*X*^2^ = 264.51, *p* < 0.001), and moving (*X*^2^ = 100.36, *p* < 0.001), and negatively for recumbent (*X*^2^ = 102.11, *p* < 0.001) and being seated at home (*X*^2^ = 203.29, *p* < 0.001) across the momentary assessments. Very similar results were found for social and non-social interactions. The interaction appraisal average was significantly associated with the number of interactions, correlating positively with the occurrence of both social interactions (*X*^2^ = 81.46, *p* < 0.001), and nonsocial interactions, (*X*^2^ = 72.61, *p* < 0.001). PA was associated with the occurrence of social interactions (*X*^2^ = 49.58, *p* < 0.001) as well as with more non-social interactions, (*X*^2^ = 24.69, *p* = 0.016).

In the final analyses, we added treatment condition (oxytocin vs. placebo) to the analyses of the five activity variables and the mood/affect and appraisal variables, examining the interaction of oxytocin treatment condition by treatment week. While there was a significant interaction of treatment week x treatment condition for moving activity (increased moving activity with oxytocin; treatment x week: *X*^2^ (2) = 6.45, *p* = 0.04 (Table S3), there were no significant treatment x week interactions with the other four activities or the two interpersonal interaction variables (all *X*^2^ (2) < 3.42, all *p* > 0.07). For the mood/affect variable there was significant interaction of treatment x treatment week for PA (increased PA with oxytocin: *X*^2^ (2) = 6.15, *p* = 0.046) and NA (reduced NA with oxytocin: *X*^2^ (2) = 16.56, *p* < 0.001). In addition, statistically significant interactions of treatment x treatment week were seen for both warmth/trust in interactions (greater warmth/trust with oxytocin: *X*^2^ (2) = 6.72, *p* = 0.035) and competence in interactions (greater competence with oxytocin: *X*^2^ (2) = 9.85; *p* = 0.007).

## 4. Discussion

This secondary analysis examined longitudinal changes in EMA-reported sedentary and non-sedentary activities and their relationships with momentary measures of affect and interpersonal interaction appraisals in a sample of individuals with SSDs who received CBSST and either oxytocin or placebo. Overall, momentary reports of sedentary activities did not change significantly over the course of the 24-week treatment trial, thereby contradicting our hypothesis. Momentary affect and interpersonal interaction appraisals all improved significantly over time during treatment with CBSST, and longitudinal relationships between momentary affect, interactions, and specific activity domains were found. Oxytocin treatment was found to be associated with several additional benefits over CBSST alone, with increased PA and reduced NA as well as greater perceived warmth/trust and competence in interpersonal interactions above and beyond the effects of CBSST. Furthermore, there was a modest but significant effect of oxytocin on the likelihood of performing an activity involving movement. Taken together, these findings suggest that treatments such as CBSST which improve momentary affect, warmth/trust, and competence in interactions, reduce defeatist interaction appraisals, and improve certain aspects of functioning are not sufficient on their own to reduce sedentary behavior in those with SSDs, pointing to a need for adjunct interventions to address this important public health concern. Oxytocin as an adjunct treatment did have a modest impact on physical activities.

Fifty-four percent of EMA-reported activities were categorized as seated at home or recumbent, thereby suggesting high levels of sedentary behaviors. These results are consistent with the high levels of sedentary behavior and low rates of physical activity observed among persons with SSDs [1,2,3,4,31]. By definition, seated away activities require movement in order to leave the home, thereby making these activities less sedentary than those carried out while seated at home. Our participants were required to leave their homes at least twice per week for treatment. As a result, their level of seated away from home activities (11%) was likely to be considerably higher by default than naturalistic studies of participants in short-term observational studies (e.g., [14,15]). Thus, these participants manifest a much higher level of away activities than participants in previous observational studies with very similar EMA methods. For example, in the Granholm et al. (2020) study participants reported that they had been at home at least half of the last hour on 80% of their surveys, and for the whole hour on 64% of their surveys [14]. In a longer study with 90 EMA surveys over 30 days, participants with schizophrenia were at home for 66% of the time surveyed [15]. It is entirely possible that the propensity to leave their homes in order to participate in a treatment trial means that these participants are already considerably more likely to be engaging in activities away from home; hence, there may be less room for change in their daily behavior. Given the notable changes in mood associated with CBSST, it seems very plausible that an augmented intervention with even more focus on activities would lead to reductions in sedentary behavior.

Despite all participants receiving 24 weeks of CBSST, an effective group treatment for negative symptoms and functioning [19,22,23,24], there were no observed changes in any of the activity domains over time for the overall sample. These null findings may be reflective of CBSST’s greater impact on cognitive rather than behavioral domains. Specifically, CBSST has been shown to improve functioning and negative symptoms by way of modifying defeatist beliefs [32] through teaching participants how to identify, challenge, and reframe thoughts that interfere with functioning. As illustrated in our analysis, CBSST resulted in improved dysfunctional interpersonal interaction appraisals (i.e., competence and warmth/trust); however, it did not lead to significant changes in the number of social interactions or numbers of activities across domains. Therefore, it may be the case that while participants developed important cognitive skills to apply to their current level of behavioral engagement, this improvement did not lead to making significant changes to their physical or social activities. In addition, and in contrast to negative findings for in-lab measures of symptoms and functioning behaviors reported in Buchanan et al. (2021) [25], oxytocin added an incremental benefit to CBSST in term s of mood and defeatist interaction appraisals, along with a modest increase in the likelihood of having completed a moving activity in the past hour. The significant improvements found with oxytocin on EMA measures and not in-lab measures of defeatist interaction and mood appraisals may suggest that EMA measures are more sensitive to these outcomes.

Momentary affect improved over the 24 weeks, with participants reporting greater PA and less NA. Further, PA was associated with greater proportions of standing, moving, and seated away from home activities and lower proportions of recumbent and seated at home activities. These relationships are generally consistent with EMA research studies of people without SSDs, which have shown relationships between PA and reduced sedentary behavior and greater physical activity [33,34,35]. Our finding that PA was related to greater seated away from home activities may illustrate the importance of context when considering the relationship between affect and activity. In fact, one study showed that there were higher levels of EMA-reported PA when physical activity was conducted with other people compared to being alone and that lower NA was present when physical activity was conducted outdoors compared to indoors [36]. The seated away from home activities in our sample almost all included being around (or interacting with) other people at a location in the community, which contrasts with seated at home activities, which are mostly solitary behaviors. Therefore, these findings illustrate that the differential relationship between sedentary activities and PA in our sample may have resulted from the influence of contextual factors (i.e., location and presence of others).

Despite the improvements in dysfunctional social appraisals and affect as well as relationships with activity domains, our results demonstrate that changing affect and defeatist appraisals does not necessarily yield behavioral changes, suggesting a need for additional components of intervention. A number of exercise programs have been shown to be effective at increasing activity and improving fitness for those with SSDs [37,38,39]. Further, multicomponent lifestyle (e.g., those with exercise and nutrition/diet components) programs have been shown to be effective at improving fitness and promoting weight loss among individuals with SSDs [40,41,42], and could serve as a valuable intervention model for a combined CBSST and activity program. Given that CBSST improved functioning and affect, the inclusion of an additional intervention component aimed at increasing engagement in non-sedentary activities could extend this program’s impact to physical health and lead to participants having a positive reaction to these increases in activity. Furthermore, the activity component could be used to facilitate greater participation in social activities as a means for individuals to practice social communication skills. By addressing social functioning difficulties, one possible underlying reason for high sedentary behavior in this population, this type of combined program could result in sustained behavior change.

Several limitations should be considered when interpreting the results of this secondary analysis. First, because participants did not report their mode of movement for each activity it is possible that our categorization of activities did not accurately reflect the type of movement involved in completing all activities for all participants. Second, there was no comparison condition of participants who did not receive CBSST (i.e., all participants in the sample received CBSST), which precludes conclusions as to any changes that would have occurred solely with the passage of time. Third, although the EMA design confers many strengths (e.g., greater number of assessments, momentary measures), all items were self-reported and are subject to potential self-reporting inaccuracies. Despite these limitations, this study offers one of the first longitudinal examinations of real-world sedentary behavior in individuals with SSDs. These findings underscore the need for novel and targeted strategies to increase activity and reduce sedentary time in this population.

## Figures and Tables

**Table 1:** Categorization of EMA-reported Activities.

Recumbent	Seated at Home	Seated Away from Home	Standing	Moving
Lay down and rest	▪ Watch TV ▪ Sit alone ▪ Work on arts and crafts/hobby ▪ Pay bills/plan budget ▪ Read a book, magazine, or newspaper ▪ Use the internet or computer ▪ Play cards/games ▪ Look for work ▪ Do school homework ▪ Listen to music/radio ▪ Play a musical instrument	▪ Go to cinema, theater, or sporting event ▪ Attend a class ▪ Go to a doctor ▪ Go to therapy, counseling, or group ▪ Attend a support group ▪ Meet with case manager or social worker ▪ Eat out	▪ Cook or prepare food ▪ Change clothes/get dressed ▪ Brush teeth (or dentures) ▪ Use ATM or bank services ▪ Groom (hair, nails, etc.) ▪ Shower or bathe	▪ Exercise, walk, play sports ▪ Clean your house or room ▪ Shop for groceries ▪ Wash, dry, fold, sort laundry ▪ Visit a beach or park ▪ Work at job program or volunteer job ▪ Work paid job ▪ Attend religious service/activity ▪ Visit a clubhouse or community center ▪ Hang out or pace

**Table 2:** Baseline Scores on Activity Domains and Interaction Variables.

	*M*	*SD*	*N*
*Activity Domain Proportions*			
Recumbent	0.15	0.27	1251
Seated at Home	0.39	0.35	1251
Seated Away from Home	0.11	0.26	1576
Standing	0.18	0.27	1576
Moving	0.17	0.28	1576
*Number of Interactions*			
Social	1.14	1.18	1203
Non-Social	0.59	0.83	1203

Note. *M* = mean, *SD* = standard deviation. Values represent averages of aggregated scores (within participant) across up to seven surveys per day for up to seven days during baseline.

**Table 3:** Time Course of Affect States and Interaction Appraisals.

	PA		NA		Warmth/Trust		Competence	
	*X* ^2^	*p*	*X* ^2^	*p*	*X* ^2^	*p*	*X* ^2^	*p*
Omnibus	112.92	<0.001	50.91	0.002	101.28	<0.001	97.12	<0.001
Intercept	52,080.62	<0.001	12,512.34	<0.001	30,433	<0.001	44,392	<0.001
Time of Day	32.22	<0.001	9.35	0.16	12.89	0.045	8.38	0.21
Day	4.80	0.57	5.84	0.48	17.56	0.007	10.93	0.091
Week	48.09	<0.001	15.70	<0.001	57.14	<0.001	65.98	<0.001
EM Means	PA		NA		Warmth/Trust		Competence	
	*M*	*SEM*	*M*	*SEM*	*M*	*SEM*	*M*	*SEM*
Baseline	9.28	0.07	5.32	0.07	4.91	0.05	5.42	0.04
Week 12	9.82	0.08	4.92	0.08	5.29	0.05	5.76	0.05
Week 24	9.93	0.08	5.00	0.08	5.42	0.05	5.94	0.05

Note. PA = positive affect; NA = negative affect; *M* = mean; *SEM* = standard error of the mean.

## Data Availability

The data presented in this study are available on request from the corresponding author. The data are not publicly available to protect participant privacy.

## Supplementary Materials

The following supporting information can be downloaded at: https://www.mdpi.com/article/10.3390/bs12030060/s1, Table S1: Sample Demographics of Parent RCT; Table S2: Time Course of EMA-reported Activities and Interactions; Table S3: Significant Interactions of Treatment (oxytocin v. placebo) x Week for EMA-reported Activities, Mood/Affect, Interactions, and Interpersonal Interaction Appraisals.
