# Supplementary material for: A Longitudinal Examination of Real-World Sedentary Behavior in Adults with Schizophrenia-Spectrum Disorders in a Clinical Trial of Combined Oxytocin and Cognitive Behavioral Social Skills Training
Source: Behav Sci (Basel). 2022 Feb 23;12(3):60. doi: 10.3390/bs12030060 (PMC8945120; doi:10.3390/bs12030060)
Supplement: Supplementary file 1 [file behavsci-12-00060-s001.zip › behavsci-1582146-supplementary.pdf]

Table S1. *Sample Demographics of Parent RCT*

	**CBSST + Oxytocin (*n*=31)**	**CBSST + Placebo (*n*=31)**
Age (years)	42.8 (8.7)	40.7 (10.2)
Sex		
Male	18 (58.0)	20 (64.5)
Female	13 (42.0)	11 (33.5)
Race		
White	17 (54.8)	15 (48.4)
Black	9 (29.0)	7 (22.6)
Other	5 (16.1)	7 (22.6)
Education (years)	13.0 (1.9)	13.4 (2.3)

Note. The present study included the 57/62 participants that completed any EMA surveys.

Table S2. *Time Course of EMA-reported Activities and Interactions*

	**Recumbent**		**Standing**		**Moving**		**Seated Home**	
	X^2	p	X^2	p	X^2	p	X^2	p
Omnibus	156.628	<.001	133.329	<.001	39.810	<.001	322.725	<.001
Intercept	109.400	q	213.866	<.001	203.941	<.001	3121.248	<.001
Week	1.148	.563	4.230	.121	1.745	.418	3.929	.269
Day	21.316	.002	8.930	.178	22.423	.001	30.104	<.001
Time of Day	138.990	<.001	122.681	<.001	15.609	.016	291.211	<.001

	**Seated Away**		**Social Interactions**		**Non-Social Interactions**	
	X^2	p	X^2	p	X^2	p
Omnibus	358.920	<.001	141.138	<.001	151.956	<.001
Intercept	496.955	<.001	556.027	<.001	3.303	.069
Week	4.454	.216	1.407	.495	8.816	.012
Day	113.260	<.001	20.373	.002	46.099	<.001
Time of Day	250.560	<.001	59.179	<.001	24.129	<.001

Table S3. *Significant Interactions of Treatment (oxytocin v. placebo) x Week for EMA-reported Activities, Mood/Affect, Interactions, and Interpersonal Interaction Appraisals*

**Outcome**	$\boldsymbol{X^2}$	**df**	***p***
Moving Activities	6.45	2	.040
PA	6.15	2	.046
NA	16.56	2	<.001
Warmth/Trust	6.72	2	.035
Competence	9.85	2	.007

Note. PA = positive affect; NA = negative affect. The table above provides statistics for significant interactions of treatment*week. Treatment*week interactions for the remaining outcomes (social interactions, non-social interactions, standing activities, recumbent activities, and total seated activities) were not significant ($X^2(2) < 3.42$, all $p > .07$).
